# Comparison of transcriptome profiles between medulloblastoma primary and recurrent tumors uncovers novel variance effects in relapses

**DOI:** 10.1186/s40478-023-01504-1

**Published:** 2023-01-12

**Authors:** Konstantin Okonechnikov, Aniello Federico, Daniel Schrimpf, Philipp Sievers, Felix Sahm, Jan Koster, David T. W. Jones, Andreas von Deimling, Stefan M. Pfister, Marcel Kool, Andrey Korshunov

**Affiliations:** 1 Hopp Children’s Cancer Center Heidelberg (KiTZ), Heidelberg, Germany; 2 Division of Pediatric Neuro-Oncology, German Cancer Research Center (DKFZ), German Cancer Consortium (DKTK), Heidelberg, Germany; 3 Clinical Cooperation Unit Neuropathology, German Cancer Research Center (DKFZ), German Cancer Consortium (DKTK), Heidelberg, Germany; 4 Department of Neuropathology, Heidelberg University Hospital, Heidelberg, Germany; 5 Center for Experimental and Molecular Medicine, Amsterdam University Medical Centers, University of Amsterdam and Cancer Center Amsterdam, Amsterdam, The Netherlands; 6 Division of Pediatric Glioma Research, German Cancer Research Center (DKFZ), Heidelberg, Germany; 7 Department of Pediatric Hematology and Oncology, Heidelberg University Hospital, Heidelberg, Germany; 8 Princess Máxima Center for Pediatric Oncology, 3584 CS Utrecht, The Netherlands

**Keywords:** Medulloblastoma, Relapses, Transcriptomics, Prognosis

## Abstract

Nowadays medulloblastoma (MB) tumors can be treated with risk-stratified approaches with up to 80% success rate. However, disease relapses occur in approximately 30% of patients and successful salvage treatment strategies at relapse remain scarce. Acquired copy number changes or *TP53* mutations are known to occur frequently in relapses, while methylation profiles usually remain highly similar to those of the matching primary tumors, indicating that in general molecular subgrouping does not change during the course of the disease. In the current study, we have used RNA sequencing data to analyze the transcriptome profiles of 43 primary-relapse MB pairs in order to identify specific molecular features of relapses within various tumor groups. Gene variance analysis between primary and relapse samples demonstrated the impact of age in SHH-MB: the changes in gene expression relapse profiles were more pronounced in the younger patients (< 10 years old), which were also associated with increased DNA aberrations and somatic mutations at relapse probably driving this effect. For Group 3/4 MB transcriptome data analysis uncovered clear sets of genes either active or decreased at relapse that are significantly associated with survival, thus could be potential predictive markers. In addition, deconvolution analysis of bulk transcriptome data identified progression-associated differences in cell type enrichment. The proportion of undifferentiated progenitors increased in SHH-MB relapses with a concomitant decrease of differentiated neuron-like cells, while in Group 3/4 MB relapses cell cycle activity increases and differentiated neuron-like cells proportion decreases as well. Thus, our findings uncovered significant transcriptome changes in the molecular signatures of relapsed MB and could be potentially useful for further clinical purposes.

**Supplementary Information:**

The online version contains supplementary material available at 10.1186/s40478-023-01504-1.

## Introduction

During the past decades, progress in the treatment of medulloblastoma (MB) has resulted in an increased 5-years overall survival of up to 80% for standard-risk patients [17]. Unfortunately, in 30% of MB patients tumors may re-occur with limited options for curative treatments and survival rates after relapse being low, but varying between patients’ groups. Some infants with SHH-MB typically have longer post-relapse survival, while treatment of older children has a low success rate despite the integration of multi-modal treatment approaches [11, 12]. The location of relapse tumors has specific patterns: in approximately 30% of the cases, relapses are located in the same region as the original tumors, while in another 50% of the cases relapses occur distantly and tumors can metastasize to different central nervous system regions. In around 20% of the cases, tumors relapse both locally and distantly [18, 26]. In general, recurrent tumors remain resistant to existing therapies and no optimal established treatment strategies for MB relapses exist so far [6, 10].

Since 2016, the WHO has included a classification of MB based on molecular profiles. Initially, four consensus MB molecular groups were outlined: WNT-MB, SHH-MB, Group 3 MB and Group 4 MB [25]. In 2021, in the 5th WHO edition of the classification of CNS tumors, a refined molecular classification has been adopted; this update includes a split of SHH-MB into four subgroups [7] and Group 3/4 MB (also termed as non-SHH/non-WNT) into eight subgroups, each with distinct genetic and clinical characteristics [23]. This molecular classification of MB has improved diagnostics risk stratification and becomes more integrated as a standard procedure for patient inclusion in clinical trials as well as other decisions on treatments [3]. However, an important research question remains how various molecular properties of the tumors may change upon relapse in order to adjust the optimal treatment strategy.

Since understanding such molecular differences of relapse tumors is critical in order to develop more effective therapies, several studies have comparatively investigated primary and recurrent MB tumors [14, 19]. In particular, it has been shown that the main variance in genomic changes are the unique additional driver mutations (covering around 41% of relapses) and acquired somatic DNA alterations (approximately 53% of relapses) occurring in the recurrent MB [16, 27]. At the same time, methylation profiles used to distinguish different molecular MB variants largely remained stable between primary and relapse tumors across all MB groups [14, 17, 18, 28].

In this study, we have investigated the transcriptional differences between primary and relapsed MB to find progression-associated molecular alterations. For this purpose, we analyzed a cohort of primary-relapse MB sample pairs (n = 43), covering SHH-MB, Group 3 MB and 4 MB molecular groups performing RNA-sequencing on all of them. Apart from describing transcriptional changes between primaries and relapses and analyzing differentially expressed genes for each of the molecular groups, we have also performed de-convolution analyses of the RNA sequencing profiles using published MB single-cell transcriptome data as a reference to see what happens with different cell populations within a tumor during the course of the disease.

## Methods

### Patient cohort and tumor molecular characteristics

In this study, 43 primary-relapse pairs of tumors with initial diagnosis medulloblastoma (MB), which relapsed following upfront therapy were selected from the international tumor set molecularly analyzed at the German Cancer Research Centre (DKFZ, n = 643). The study was conducted under the auspices of the local Ethics Committees, in compliance with German rules of the Health Insurance Portability. Pathological MB diagnosis and histological tumor variants were assigned according to 2021 WHO criteria [3] and metastatic status at diagnosis was determined according to Chang's system. Treatment details and follow-up data were available for all patients who were operated on and received combined treatments with HIT-based protocols as described [6, 13]. Institutional imaging reports were collated and locally reviewed by an experienced panel of neuro-oncologists to assess patterns of relapse.

Targeted exome sequencing and methylation data were generated and analyzed as described previously [13]. Tumor purity was computed from methylation profiles using the tool ESTIMATE [29], resulting in a mean ~ 90% of tumor content per sample.

All tumors were molecularly classified from the methylation data into one of the four consensus” MB molecular groups (WNT-MB, SHH-MB, Group 3 MB, and Group 4 MB) according to the “Heidelberg brain tumor classifier; v11b4” (www.molecularneuropathology.org). Tumors were also assigned to “second-generation; v12.5″ subgroups including subgroups 1–4 for MB-SHH and subgroups I–VIII for Group 3 and Group 4 MB as described. Copy number profiles were generated using the ‘conumee’ R package. Multicolor interphase fluorescence in situ hybridization (FISH) analysis for *MYC, MYCN, GLI2, CCND2* and *CDK6* DNA probes was performed for all primary and relapsed MB samples as described [14].

### RNA-sequencing data generation and analysis

RNA was extracted from formalin-fixed and paraffin-embedded (FFPE) tissue samples and RNA sequencing was performed on a NextSeq 500 (Illumina) as described [22]. The reads were aligned to hg19 reference using STAR version 2.5.2b and for each sample [4], gene expression was quantified by the feature counts module of the Subread package version 1.4.6 using Gencode version 19 annotations with considering uniquely mapped reads only [15]. Afterwards, gene expression counts were adjusted with log2 RPKM expression normalization.

Target gene expression submatrix was derived from the top 500 most highly variable genes in the selected cohort. Euclidian distance between primary and relapse cases was computed either within the combined top 3 principal components and further used as variance measure value. Statistical evidence in difference of variance between selected groups of primary-relapse pairs (e.g. local and metastasis) was measured with T-test. Differential gene expression analysis between primary and relapse cases was performed using limma R package (adjusted *p*-value < 0.05) with patient pair assignment for batch effect adjustment [21]. Gene ontology was estimated via ClueGO Cytoscape plugin [1].

Deconvolution analysis was performed with CIBERSORTx tool based on 10X protocol adjusted settings [24] using the raw gene expression count matrices of the bulk dataset and of the corresponding MB single-cell RNA-seq dataset [20] as the reference to impute the fractions of the single cell populations. We applied S-mode (single cell mode) batch correction to minimize the technical effects given by the different platforms from which signature (reference) and mixture (bulk) matrices have been generated. We set the number of permutations for statistical analysis to 100. Statistical evidence of a relative difference in cell types proportions between primary and relapse samples was measured with T-test.

In order to verify the deconvolution results, gene set variance analysis (GSVA) [9] was performed on mean gene expression values computed from RPKM matrices for primary and relapse target group sample cohorts.

### Statistics

Chi-squared and Fisher's exact tests were used to assess associations between clinical and molecular features were performed on the international MB DKFZ RNA-seq cohort (n = 643) and external Cavalli et al. [2] Affymetrix cohort (n = 377). The log-rank test was used in uni-variable analyses to assess the time from relapse to death (for tumors with relapses), and overall survival (for the entire cohort), and the Kaplan–Meier method was used to visualize results.

Survival analyses based on the expression of single genes or multiple genes were performed with special algorithms using a Bonferroni correction for multiple testing. For multivariate analysis, Cox proportional hazards regression models were used and estimated hazard ratios are provided with 95% confidence intervals. The result plots were created with R2: Genomics Analysis and Visualization Platform.

### Availability of data and materials

The dataset generated and analyzed during the current study (normalized gene expression counts matrix) is available in the R2 platform (http://r2.amc.nl) with the name “Tumor Medulloblastoma—Korshunov—86—rpkm—mbffpe”.

## Results

### Clinical-molecular characteristics of primary-relapse medulloblastoma cohort

**Fig. 1:**
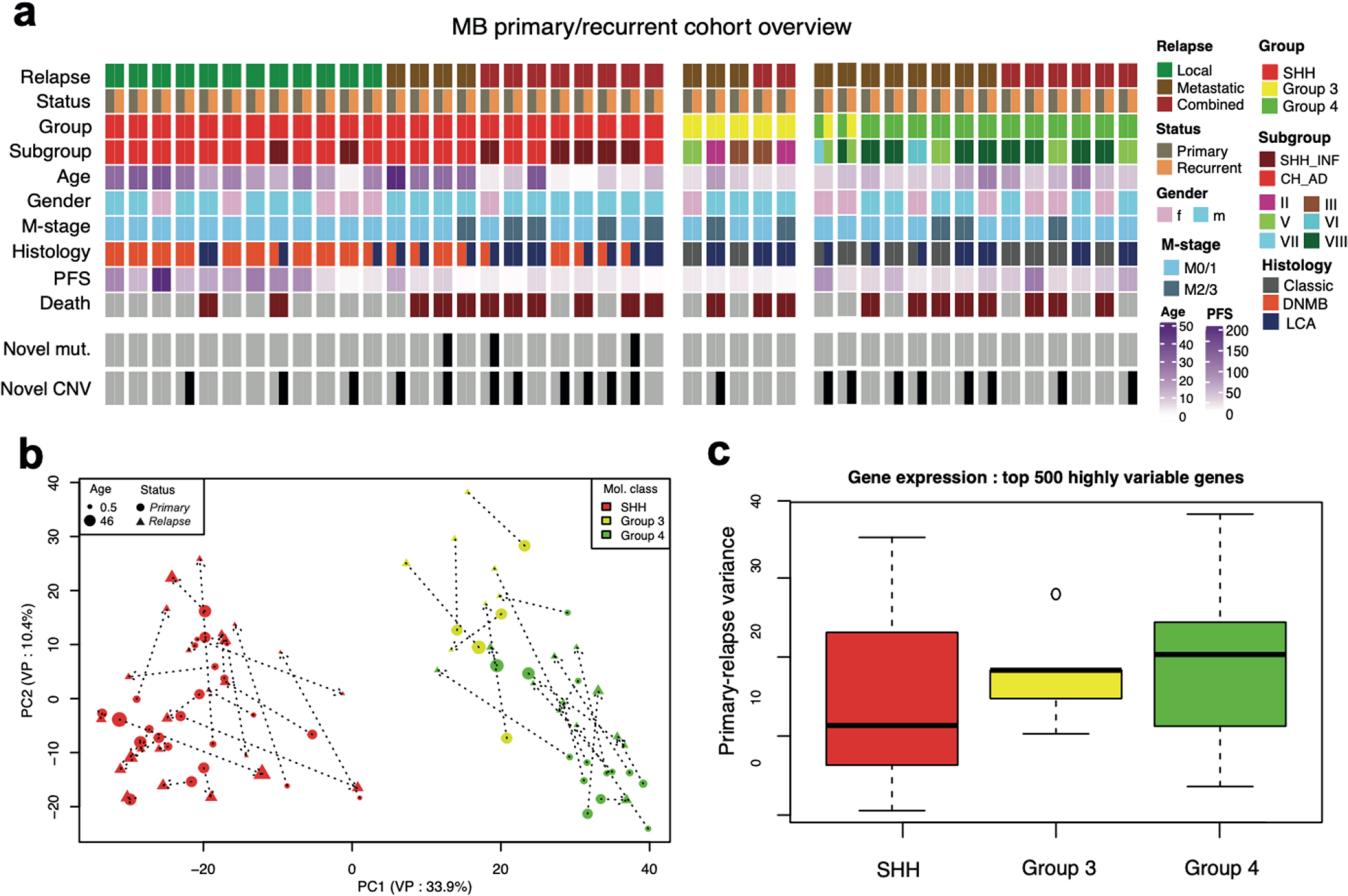
**a** Annotation onco-plot describing patient histological and molecular characteristics for target primary-relapse tumor pairs with available RNA sequencing data (n = 43). The following abbreviations were used: SHH_INF—infant SHH, CH_AD—child–adult SHH, DNMB—desmoplastic/nodular, LCA—large cell/anaplastic, PFS—progression-free survival, CNV—copy number variants. **b** Principal component analysis of full MB gene expression primary-relapse dataset based on the top 500 most highly variable genes. Tumor profiles from the same patient connected via dot lines, target component variance percentage (VP) is shown in axis labels. **c** Boxplot demonstrating the transcriptome variance between primary and relapse tumors among MB groups (SHH-MB—24, Group 3 MB—5, Group 4 MB—14 cases)

Forty-three primary and relapse tumor samples identified via DNA methylation profiling as MB were selected for the investigation of their paired transcriptome profiles. The cohort included 24 SHH-MB, 5 Group 3 MB and 14 Group 4 MB pairs (Fig. 1a, Additional File 2: Table S1). WNT-MB was excluded from this study due to the low number of primary-relapse sample transcriptome profiles (n = 1) available in our DKFZ cohort.

Evaluation of the radiological images (see “Methods” Section, Additional File 2: Table S1) identified anatomic patterns of relapsed MB as follows: (1) isolated local relapses (12/43 [28%] MB; SHH-MB/Group 3 MB/Group 4 MB distribution 12/0/0; mean age—17 years; male/female ratio 57%/43%; M2-3 at diagnosis—10%; median PFS—64 months); (2) distant/metastatic relapses (15/43 [35%] MB; SHH-MB/Group 3 MB/Group 4 MB distribution 4/4/7; mean age—17 years; male/female ratio 70/30%; M2-3 at diagnosis—30%; median PFS—30 months) and (3) combined local/distant relapses (16/43 [37%] MB; SHH-MB/Group 3 MB/Group 4 MB distribution 8/2/6; mean age—13 years; male/female ratio 70%/30%; M2-3 at diagnosis—40%; median PFS—30 months). Histopathological evaluation revealed that 32/73% MB pairs had consistent tumor histology at primary diagnosis and at relapse (among them—10 classic MB; 11 desmoplastic/nodular MB (DNMB), and 11 large-cell/anaplastic MB (LCA)), while 12/27% MB primaries disclosed histological diversity at the relapse when 3/13 classic MB (23%) and 9/20 DNMB (45%) were diagnosed as LCA at relapse. There was no preponderance of anatomic relapse patterns for tumors with and without histological divergence.

Medulloblastoma molecular groups and subgroups were conserved at relapse in 41 (95%) cases and only two Group 4 MB primary tumors demonstrated a switch to Group 3 MB at relapse, confirming a rarity of progression-associated MB group change that was observed previously in ~ 5% of Group 3/4 MB recurrences [14]. In these two samples, second-generation subgroups also switched from VII to V in one pair and from VIII to V in another one, respectively.

Somatic changes were identified in MB relapses with targeted exome sequencing and DNA methylation profiling (see “Methods” Section; Additional File 2: Table S1). Thus, 3 relapsed MB (7%) demonstrated variance in driver mutations whereas 20 cases (43%) disclosed differences in copy number changes. For example, *TP53* somatic mutations were identified as relapse-specific in two infant SHH *TP53*-wt tumors (both harboring *PTCH1* germline mutations). Relapse-specific amplification of *MYCN* was identified from methylation and FISH data (see Methods) in one SHH-MB and one Group 4 MB, similarly one SHH-MB case demonstrated *GLI2* amplification only at relapse. Additional chromosomal gains and losses were identified specifically at relapse in 13/24 SHH-MB (55%), 1/5 Group 3 MB (20%) and 8/14 Group 4 MB (55%). Notably, prototypic chromosomal events such as 9q loss in SHH-MB and isochromosome 17q in Group 3 and Group 4 MB were conserved between diagnosis of primary and relapse in all affected cases.

After the concordance between primary and relapse RNA-sequencing profiles was verified based on the fingerprint SNV match (Additional File 1: Fig. S1a), we focused on the transcriptome variance analysis between primary and relapsed tumors. The gene expression data clearly demonstrated group specificity from unsupervised Principal Component (PC) analysis (Fig. 1b). The variance between primary and relapse cases was measured as Euclidian distance between top PC components (Fig. 1c), which revealed that this effect is quite strong, especially in Groups 3 and 4 MB. Notably, the PC variance in methylation data appeared to be much lower in these MB groups in comparison to gene expression (Additional File 1: Fig. S1b, c), probably driven by strong stability of this epigenetic effect [5].

### SHH-MB tumors demonstrate strong changes in relapse transcriptome profiles at young age

**Fig. 2:**
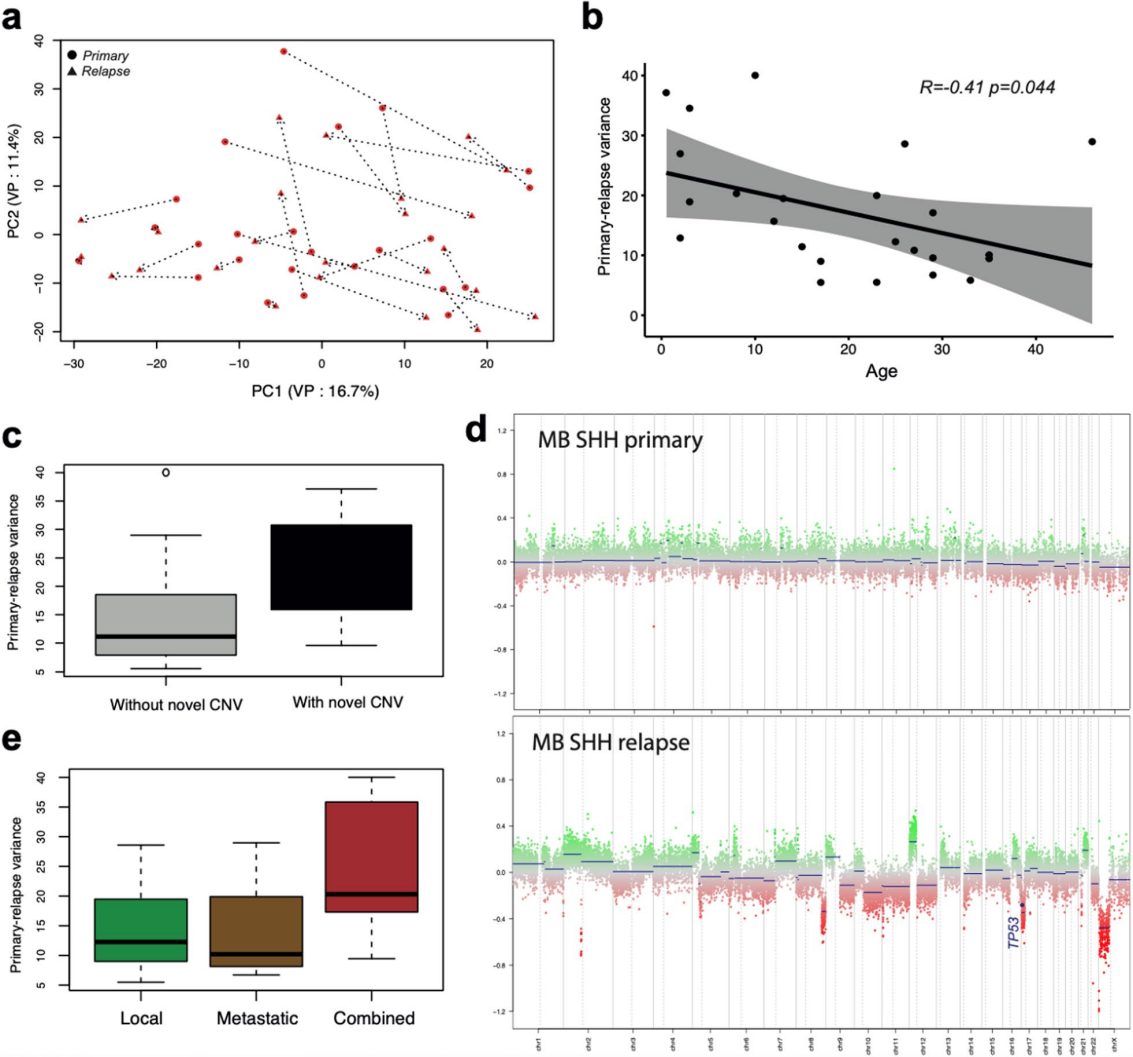
**a** Principal component analysis of SHH-MB gene expression primary-relapse dataset based on the top 500 most highly variable genes. Primary and relapse tumor profiles from the same patient connected via dot lines, target component variance percentage (VP) is shown in axis labels. **b** Association of transcriptome variance between primary and relapse SHH-MB with age of the patients. **c** Boxplot demonstrating the transcriptome variance between primary and relapse tumors among SHH-MB with (n = 11) and without (n = 13) novel CNVs. **d** Copy number profiles derived from methylation data of primary (top) and relapse (bottom) tumors from the same SHH-MB infant patient. **e** Boxplot demonstrating the transcriptome variance between primary and relapse MB SHH among relapse types (local: 12, metastatic: 4, combined: 8 cases)

As a next step, we focused on a more precise investigation of molecular groups starting from SHH-MB. The variance remained quite stable from the PC inspection within the SHH-MB cohort confirming its’ group-specificity (Fig. 2a). Interestingly, an evident negative correlation (*p*-value = 0.044) was identified between the variance of expression profiles and the age of patients (Fig. 2b). From further detailed inspection, we found that the transcriptome variances were also significantly higher for tumors with new CNVs (n = 11) as compared to those without them (n = 13) (Fig. 2c). Notably, CNV profiles occurring in relapsed SHH-MB in younger than 10 years (n = 8) demonstrate clear differences from their predominantly balanced primaries (Additional File 1: Fig. S2a) with frequent involvement of the *TP53* locus at 17p (Fig. 2d). In contrast, in SHH-MB older than 10 years (n = 16), CNV profiles typically remain stable at relapse (Additional File 1: Fig. S2b). We also identified that primary-relapse expression variance was significantly higher for combined relapses (n = 8) as compared to local (n = 12) and distant (n = 4) relapses of SHH-MB (Fig. 2e).

Further, we analyzed if there were evident differentially expressed genes (DEGs) between primary and relapse in SHH-MB. The batch-effect adjusted analysis resulted in 484 genes whose expression was highly active in relapses and 184 genes whose expression was significantly lower in relapses (Additional File 2: Table S2). Gene ontology (GO) analysis demonstrated that genes over-expressed in relapsed SHH-MB were associated with molecular processes such as N-Glycan biosynthesis, ECM-receptor interaction, and ribosome biogenesis pathways, while genes under-represented in relapses were connected to MAPK, HIF1 and neurotrophin signaling (Additional File 2: Table S3).

Next, we analyzed the clinical relevance for top DEGs overexpressed in primary (top 20) and relapsed tumors (top 20) within two extended cohorts of primary SHH-MB included in international tumor sets (see Methods): relapsed (n = 98) and entire (n = 188) (Additional File 1: Fig. S2c). However, univariate survival analysis disclosed no associations of expression of any of these DEGs with clinical outcomes for both these SHH-MB cohorts. We also inspected if there are any effects associated with the age by separately calling DEGs for infant and older patient groups, but no associations to clinical outcomes were found.

### Novel potential gene markers identified for relapse of group 3/4

**Fig. 3:**
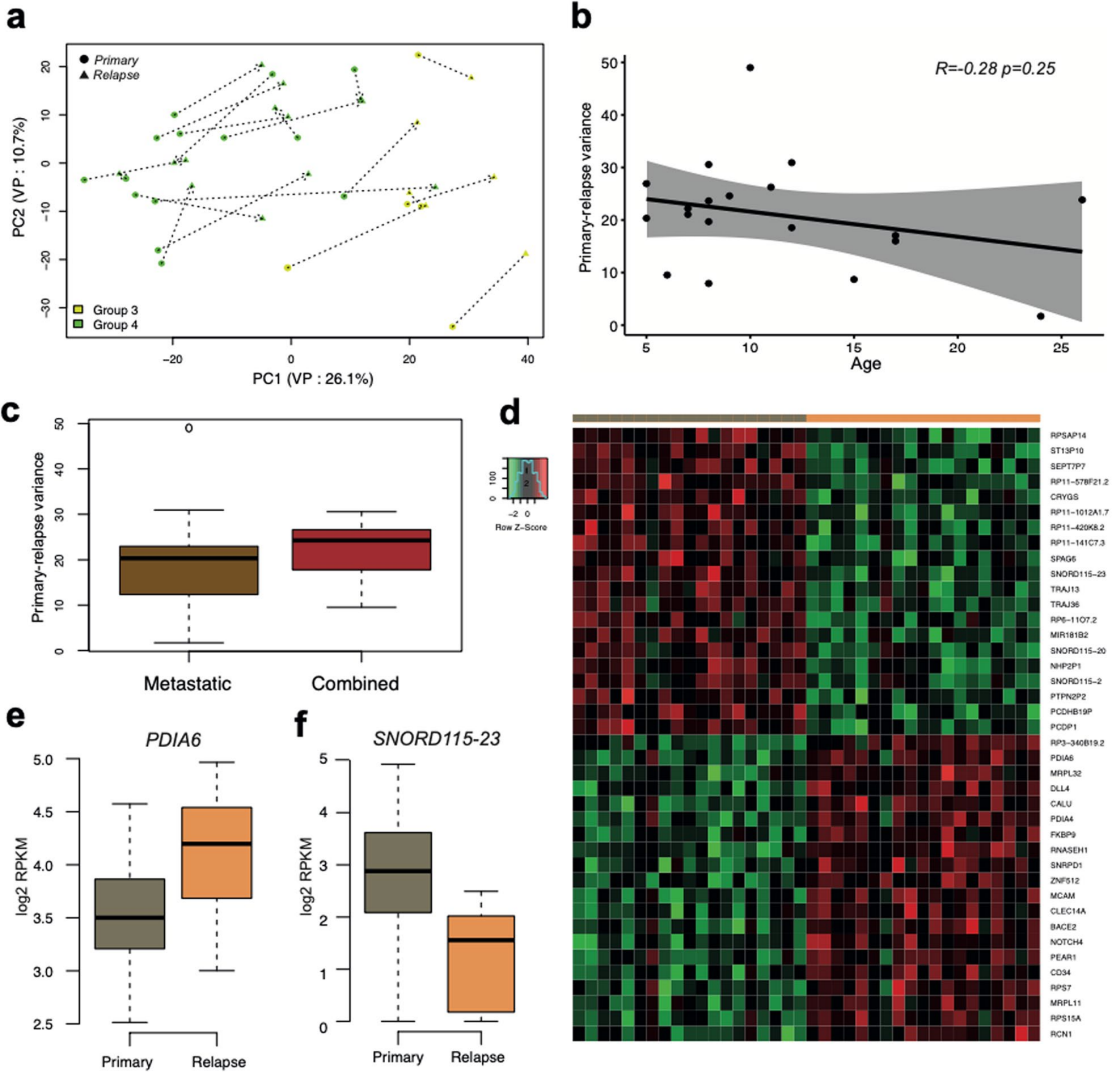
**a** Principal component analysis of Group 3/4 MB gene expression primary-relapse dataset based on top 500 most highly variable genes, target component variance percentage (VP) is shown in axis labels. Primary and relapse tumor profiles from the same patient are connected via dot lines. **b** Association of transcriptome variance between primary and relapse Group 3/4 MB with age of the patients. **c** Boxplot demonstrating the transcriptome variance between primary and relapse Group 3/4 MB among relapse patterns (metastases: 11, combined: 9 cases). **d** Heatmap of top most confident genes differentially expressed between primary and relapse Group 3/4 MB, either down-regulated (first block, n = 20) or up-regulated (second block, n = 20) in relapses respectively. **e**, **f** Boxplots of differentially expressed genes either up-regulated (**e**, PDIA6) or down-regulated (**f**, SNORD115-23) in Group 3/4 MB relapses vs primaries

We further performed a similar analysis of primary and relapse transcriptome profile differences for Groups 3 and 4 MB, merged as non-SHH/non-WNT MB, termed further as Group 3/4 MB cohort (Fig. 3a). In contrast to SHH-MB, these tumor variants disclosed no clear transcriptome variances associated with the patients’ age (Fig. 3b) or tumor relapse patterns of metastatic (n = 11) versus combined (n = 8) (Fig. 3c).

Even though some acquired CNV changes were observed in metastatic Group 3/4 MB relapses, the DNA profiles remained mostly stable for primary-relapse pairs (Additional File 1: Fig. S3a, b). In addition, the variance between primary and relapse transcriptome profiles remained quite stable in this context too, and was not associated with novel CNVs (Additional File 1: Fig. S3c).

Afterwards, we focused on the differences between primary and relapse profiles by identification of DEGs (Additional File 2: Table S4). The total amount of Group 3/4 MB DEGs (n = 585 higher expressed, n = 1526 lower expressed at relapse) was approximately three times higher in comparison to SHH-MB and more possible GO associations were identified for these gene sets (Additional File 2: Table S5). The genes overexpressed in relapsed Group 3/4 MB were found to be involved in several molecular pathways, including cell cycle activation, phagosome biogenesis, focal adhesion, and others. In contrast, genes low expressed in relapsed tumors were contributing to protein digestion, retinol metabolism, and chemical carcinogenesis.

**Fig. 4:**
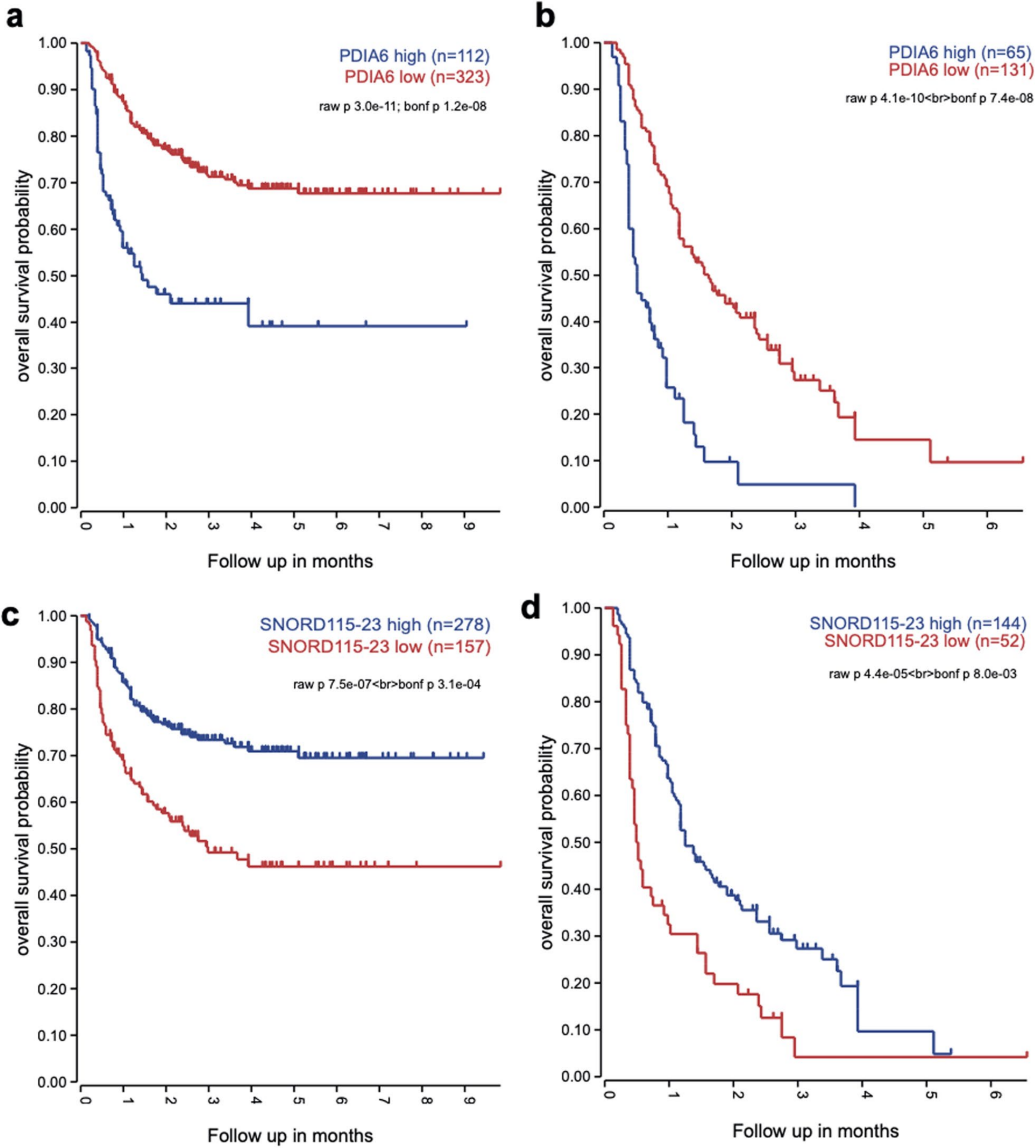
**a**, **b** Kaplan–Meyer overall survival probability curves for cases from DKFZ RNA-seq dataset with high and low expression of PDIA6 in entire (**a**) and relapsed (**b**) Group 3/4 MB cohorts disclosed unfavorable OS for tumors with elevated gene expression (log rank; *p* < 0.01). **c**, **d** Kaplan–Meyer survival probability curves for cases with high and low expression of SNORD115-23 in entire (**c**) and relapsed (**d**) Group 3/4 MB cohorts disclosed unfavorable OS for tumors with low levels of gene expression (log rank; *p* < 0.01). For relapsed cohort (b,d) survival time was calculated from re-operation to the last event

Next, we analyzed the clinical relevance for top DEG overexpressed in primary (top 20) and relapsed (top 20) tumors within two extended cohorts of primary Group 3/4 MB included in international tumor sets (see Methods): relapsed (n = 196) and entire (n = 435) (Fig. 3d). The univariate survival analysis showed that high levels of several top evident genes overexpressed in relapsed tumors such as *PDIA6* (Figs. 3e, 4a, b), *MRPL32* (Additional File 1: Fig. S4c, d) or *FKBP9* (Additional File 1: Fig. S4c, d) were associated with unfavorable PFS and OS in both relapsed and the entire Group 3/4 MB cohorts. In contrast, high expression of top genes down-regulated in relapsed Group 3/4 MB such as *SNORD115-23* (Figs. 3f, 4c, d), *TMEM261P1* (Additional File 1: Fig. S4e, f) or *RIT2* (Additional File 1: Fig. S4g, h) were associated closely with favorable outcomes for two these cohorts of Group 3/4 MB. Moreover, these results were confirmed from analysis of the external MB transcriptome dataset [2], where the same survival patterns were observed in the entire Group 3/4 MB cohort (n = 377) for *PDIA6* (Additional File 1: Fig. S5a), *FKBP9* (Additional File 1: Fig. S5b), *SNORD115-23* (Additional File 1: Fig. S5c) and *RIT2* (Additional File 1: Fig. S5d) genes.

In addition, performing differential gene expression analysis on Group 3 MB and Group 4 MB primary-relapse cohorts separately verified the marked genes independently, even though less statistical evidence was observed for Group 3 MB, probably due lower number of primary-relapse pairs in this cohort (n = 5).

Finally, we performed multigene survival analysis in relapsed Group 3/4 MB cohort (see Methods) focusing on top 20 genes differentially expressed between primary and relapsed tumors respectively (Fig. 3d). Thus, we identified that 9 genes overexpressed in relapses were significantly associated with unfavorable OS in relapsed cohort whereas 10 genes specific for primary Group 3/4 MB were associated with favorable outcomes. We combined these clinically relevant genes in two metagene cohorts—unfavorable and favorable respectively which, in turn, were correlated closely with OS of relapsed Group 3/4 MB (Additional File 1: Fig. S5 e, f).

Cox regression analysis combining clinical and molecular variables in Group 3/4 MB revealed an independent prognostic significance of outlined unfavorable metagene set together with advanced M stages and *MYC* amplification (Additional File 2: Table S6). Interestingly, molecular MB groups did not reach an independent level in this multivariate model.

### Deconvolution of bulk profiles uncovers functional structure variances between primary and relapse tumors

**Fig. 5:**
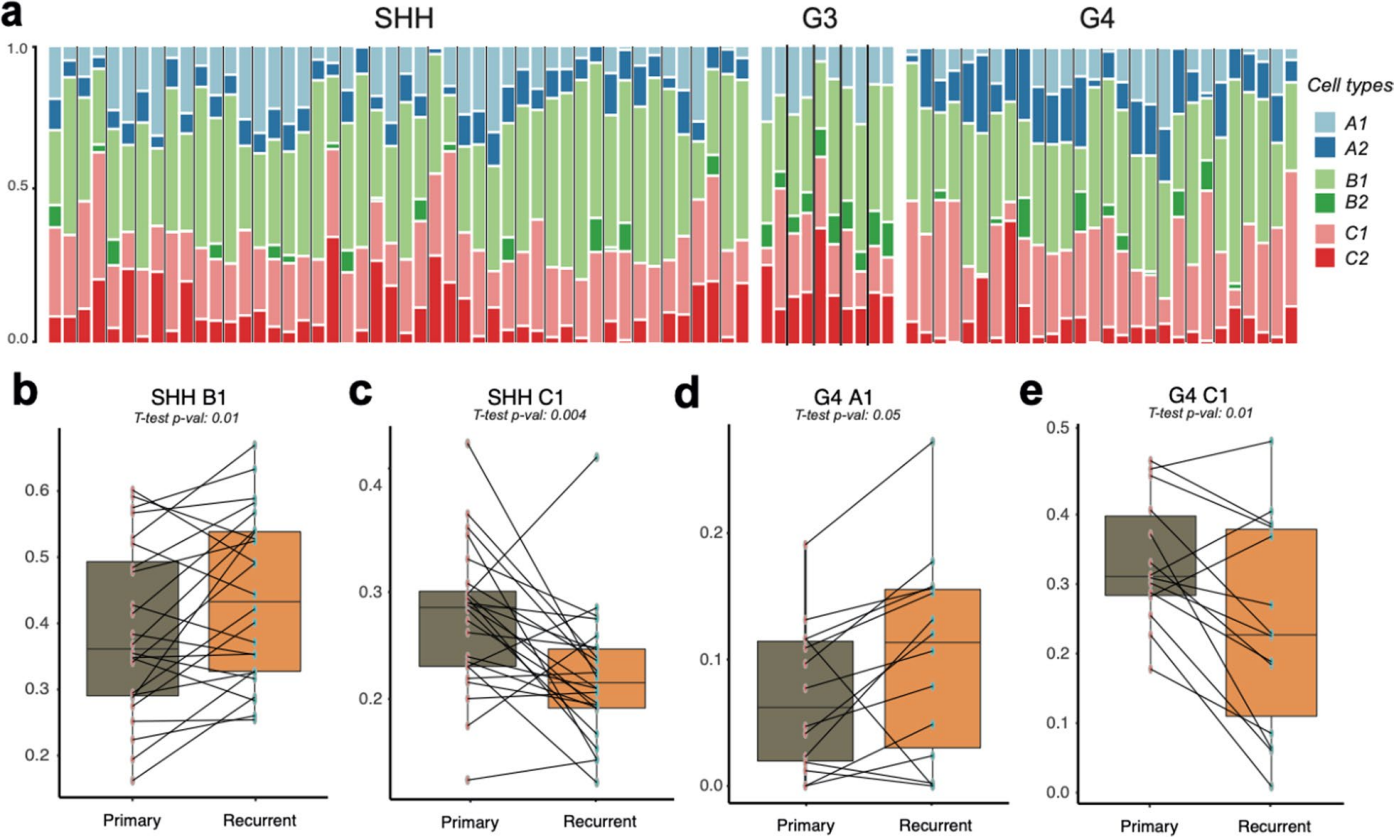
**a** Barplot demonstrating predicted relative proportions of MB cell types in bulk primary and relapse tumor gene expression profiles. Thick black lines delimitated each primary-relapse pair. **b**, **c** Boxplots of difference between MB SHH primary and relapse tumors in proportions of undifferentiated progenitors B1 (**b**) and differentiated neuron-like cells C1 (**c**). **d**, **e** Boxplots of difference between MB G4 primary and relapse tumors in proportions of cell cycle enriched A1 (**d**) and differentiated neuron-like cells C1 (**e**)

Single-cell sequencing techniques allow to understand the tumor cell composition and available single-cell profiles can serve as a reference control for analysis of bulk transcriptome profiles with deconvolution. To integrate this technique, we used as the reference a recently published single-cell profile dataset from MB [20] with detailed annotation of cell composition within them. In particular, the MB tumor cell types were distinguished into three main groups with each of them split into two subtypes: (A1; A2) cell cycle activity enriched, (B1; B2) undifferentiated progenitors and (C1; C2) differentiated neuronal-like cells. Application of the CIBERSORTx deconvolution software tool (see “Methods” section) to bulk primary and relapse transcriptome profiles demonstrated the enrichment of each cell group and subtype in a tumor at diagnosis and relapse respectively (Fig. 5a). We then analyzed group-specific differences in these compositions between primary and relapse cases. Groups 3 and 4 MB were processed separately since the reference single-cell gene profiles were provided in this format [20]. SHH-MB did not show any evident differences in cell cycle subtypes between primary and relapse samples (Additional File 1: Fig. S6a). However, the proportion of the undifferentiating progenitors appeared to be clearly increased (Fig. 5b; T-test *p*-value: 0.01), while formed differentiated neuron-like population decreased (Fig. 5c; T-test *p*-value: 0.004) in SHH-MB relapses. Notably, this effect was more evident in adult SHH-MB relapses (Additional File 1: Fig. S6b; T-test *p*-values: 0.01 in adults, 0.17 in infants).

The increased proportion of activated progenitors in SHH-MB relapses was in concordance with the GO enrichment of N-Glycan biosynthesis and ribosomal biogenesis processes, that were identified in relapse-associated DEG sets (Additional File 2: Table S3). Moreover, the effect was also inspected using gene set variance analysis (GSVA) procedure: the enrichment patterns of the corresponding MB SHH cell types in primary-relapse bulk profiles fully reflected the deconvolution results (Additional File 1: Fig. S5c).

In MB Group 4 the deconvolution was showing approximately a 1.5 times increase in cell cycle activity proportion subtype in relapses (Fig. 5d; T-test *p*-value 0.05), as was also confirmed by GO analysis (Additional File 2: Table S5). Respectively, the proportion of differentiated neuronal-like cells in the relapses vise-a-verse decreased (Fig. 5d; T-test *p*-value: 0.01), while undifferentiating progenitors did not demonstrate any evident changes between primary and relapse tumors (Additional File 1: Fig. S6d; T-test *p*-value: 0.34). In Group 3 MB the increase of cell cycle and loss of differentiated neuronal cell proportions were reflecting patterns similar to Group 4 MB (Additional File 1: Fig. S6e–g), however, the statistical evidence of the difference between primary and relapse proportion results did not pass filtering limits (*p*-value < 0.05), most likely due to low amount of input sample pairs (n = 5). Nevertheless, the results were also verified via GSVA applied for Group 4 cell type markers in primary-relapse bulk profiles (Additional File 1: Fig. S5h).

## Discussion

Even though the molecular profiles of recurrent MB cases were already studied precisely on the genomic and DNA methylation level [14, 19], the transcriptome investigations performed in this study uncovered some novel relapse-associated molecular events thus opening up new ways in our understanding of the biology of posttreatment MB progression. We identified transcriptome differences between primary and relapsed MB as well as specific genes that were associated with clinical variables (Fig. 4, Additional File 1: Figs. S4, S5), therefore may be considered as possible diagnostic targets.

For SHH-MB, the age was the main pattern correlated negatively with variance between primary and relapse gene expression profiles, which, in turn, was associated with novel genomic changes. In particular, most of the younger SHH-MB in our cohort were manifested as early combined relapses accompanied by numerous progression-associated CNVs and maximal transcriptome variability between primary and recurrent tumors. Most of these combined relapses harbored acquired 17p loss (accompanied by new *TP53* mutation in a few cases) thus suggesting bi-allelic gene inactivation with further accumulation of genomic changes.

In turn, SHH-MB from older patients (especially adults) manifested typically as late isolated local recurrences with infrequent novel CNVs and lower levels of transcriptome variability between primaries and relapses. Perhaps, variability in HIT treatment protocols applied for infant (intense chemotherapy [CHT] without craniospinal irradiation [CSI]) and adult (CSI and maintenance CHT) SHH-MB patients could have an impact on the progression-associated genome and transcriptome changes in this tumor group. However, the DEGs identified between primary and relapse SHH-MB were age-independent and not associated with patients’ clinical outcomes. It could be explained by well-established age-specific molecular properties of SHH-MB that keep it close to tumor development and progression [7]. Nevertheless, deconvolution of bulk RNA disclosed the tangible differences in cell composition between primary and relapse: the number of cell progenitors increased in the relapses, especially in adult SHH-MB.

In non-WNT/non-SHH MB (Group 3/4 MB) tumor recurrences were either distant or combined, all these patients were treated in the same way (CSI and maintenance CHT) and that could equalize posttreatment biological effects. Transcriptome differences were not associated with patients’ age, relapse pattern or acquired CNVs and were similar for Group 3 and 4 MB. However, sets of clinically relevant DEGs were identified and genes comparatively overexpressed within primary tumors were associated with favorable outcomes. Moreover, expression of most of these genes was significantly higher in non-recurrent Group 3/4 MB as compared to relapse. Therefore, low expression levels of these genes detected in primary Group 3/4 MB could be considered predictors of possible tumor recurrence thus suggesting an intensification of primary therapy upfront. In contrast, a set of genes overexpressed in Group 3/4 MB relapsed samples was associated closely with shortened patients’ OS after re-operation. These genes were clearly associated with cell cycle signaling pathways, in line with the data of deconvolution analysis, which demonstrated an increase of the “cell cycle” subtype in Group 3/4 MB relapses. Potentially these genes (or metagene sets) could be mostly used as prognosticators of relapsed Group 3/4 MB outcomes and/or as possible molecular targets for treatment-resistant recurrences, especially considering that the predictive effect was confirmed from two independent cohorts.

We could suggest that the progression-associated differences in transcriptome profiles and cell composition identified between primary and relapse MB might be related either to dormant small cell populations in primary tumors, clonally selected during treatment, or to mutagenic alterations acquired due to tumor progression under the pressure of intense radio-chemotherapy, but it remains unclear what is the main driving event. Important future research direction that could help to answer these questions is the single cell techniques application on paired primary and relapse MB tumors or their models, as it has been already started [8, 30], but requires further data extensions and experimental validations. Our results obtained from the analysis of paired MB bulk transcriptome profiles could serve as a useful source for the further studies focused on selection of potential prognostic markers and molecular target genes in this research area.

## Supplementary Information

**Additional file 1. Supplementary Figures.** The Word document file contains supplementary figures and their legends.

**Additional file 2**. **Supplementary Tables.** **Table S1**: Detailed annotation of target tumors cohort. **Table S2**: List of DEGs between primary and relapse cases in MB SHH filtered on min adj. p-value 0.05. **Table S3**: GO analysis of DEGs between MB SHH primary and relapse cases with a focus on KEGG annotation. **Table S4**: List of DEGs between primary and relapse cases in MB G3/4 filtered on min adj. p-value 0.05. **Table S5**: GO analysis of DEGs between MB G3/4 primary and relapse cases with a focus on KEGG annotation. **Table S6**: Results of multivariate analysis of OS for relapsed set Group 3/4 MB (n = 196).
