# Supplementary material for: Comparison of transcriptome profiles between medulloblastoma primary and recurrent tumors uncovers novel variance effects in relapses
Source: Acta Neuropathol Commun. 2023 Jan 12;11:7. doi: 10.1186/s40478-023-01504-1 (PMC9837941; doi:10.1186/s40478-023-01504-1)
Supplement: Supplementary file 1 — Additional file 1. Supplementary Figures. The Word document file contains supplementary figures and their legends. [file 40478_2023_1504_MOESM1_ESM.docx]

**SUPPLEMENTARY FIGURES**

| 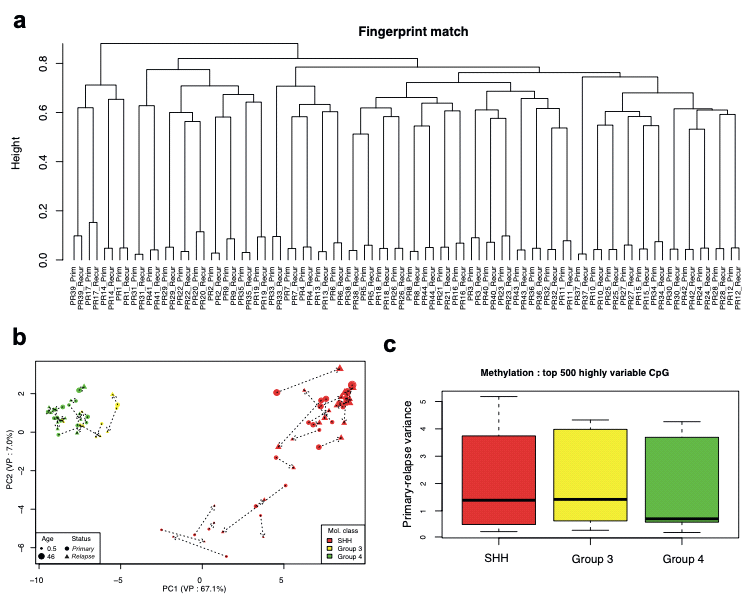 |
| --- |

***Suppl. Figure 1.*** *a) Fingerprint SNP control confirms match between primary and relapse RNA-seq profiles from the same patient. b) Principal component analysis visualization of full MB methylation dataset based on top 500 most highly CpG sites. Primary and relapse tumor profiles from the same patient connected via doted lines. c) Boxplot demonstrating the methylation variance between primary and relapse derived from top 3 principal components.*

| 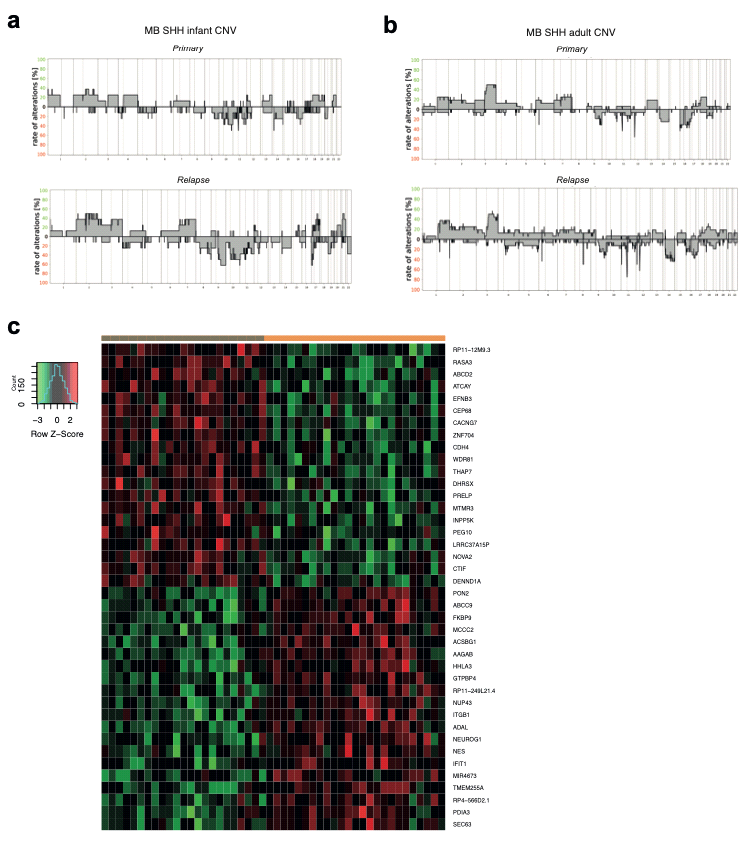 |
| --- |

***Suppl. Figure 2.*** *a) Merged CNV profiles in primary (top) and relapse (bottom) cases of SHH-MB infant group. b) Merged CNV profiles in primary (top) and relapse (bottom) cases of SHH-MB adult group. c) Heatmap of top most confident genes differentially expressed between primary and relapse MB SHH cases, either down-regulated (first block, n=20) or up-regulated (second block, n=20) in relapses respectively.*

| 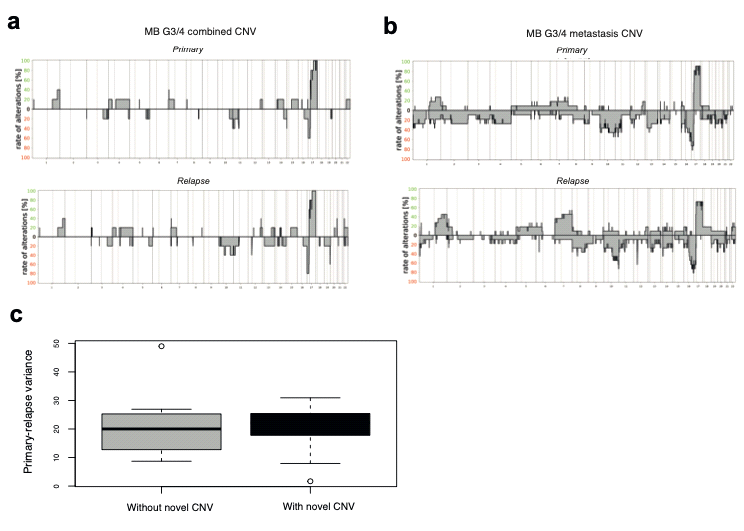 |
| --- |

***Suppl. Figure 3.*** *a) Merged CNV profiles in primary (top) and relapse (bottom) cases of Group 3/4 MB combined relapse type. b) Merged CNV profiles in primary (top) and relapse (bottom) cases of Group 3/4 MB metastasis relapse type. C) Boxplot demonstrating the transcriptome variance between primary and relapse tumors among MB G3/4 cases with (n=9) and without (n=10) novel CNVs.*

| 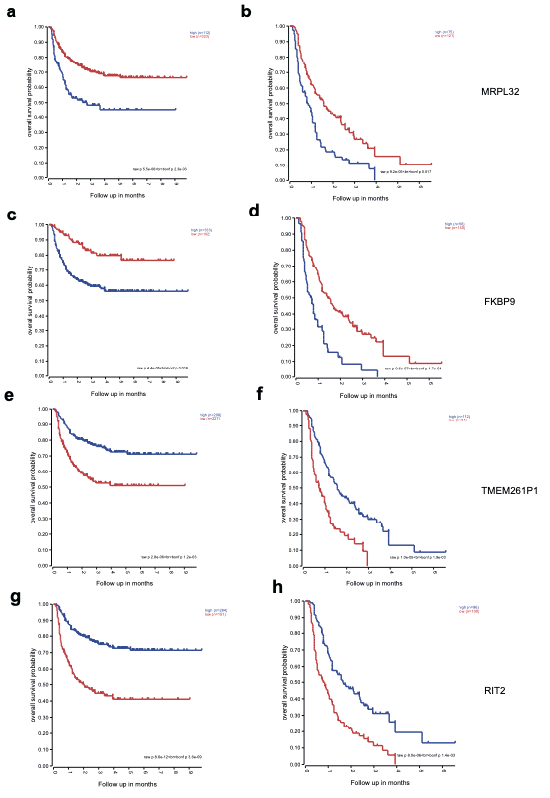 |
| --- |

***Suppl. Figure 4.*** *a,b) Kaplan-Meyer survival probability curves for cases from DKFZ RNA-seq dataset with high and low expression of MRPL32 in entire (a) and relapses (b) Group 3/4 MB cohorts c) d) Kaplan-Meyer survival probability curves for cases with high and low expression of FKBP9 in entire (c) and relapses (d) Group 3/4 MB cohorts. e,f) Kaplan-Meyer survival probability curves for cases with high and low expression of TMEM261P1 in entire (e) and relapses (f) Group 3/4 MB cohorts g,h) Kaplan-Meyer survival probability curves for cases with high and low expression of RIT2 in entire (g) and relapses (h) Group 3/4 MB cohorts. For relapsed cohort (b,d,f,h) survival time was calculated from re-operation to last event.*

| 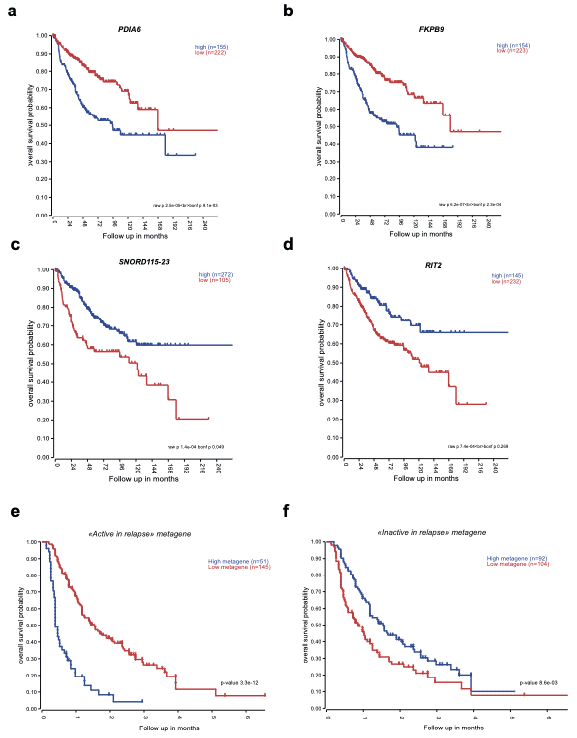 |
| --- |

***Suppl. Figure 5.*** *a,b) Kaplan-Meyer overall survival probability curves for cases from Cavalli et al. Affymetrix dataset with high and low expression for (a) PRDIA6, (b) FKPB9, (c) SNORD115-23 and (d) RIT2 in entire Group 3/4 MB cohort. e,f) Kaplan-Meyer overall survival probability curves for cases from DKFZ RNA-seq dataset with a metagene set formation of high in relapse (e) and low in relapse (f) expression for relapsed Group 3/4 MB cohort. For relapsed cohort (e,f) survival time was calculated from re-operation to last event.*

| 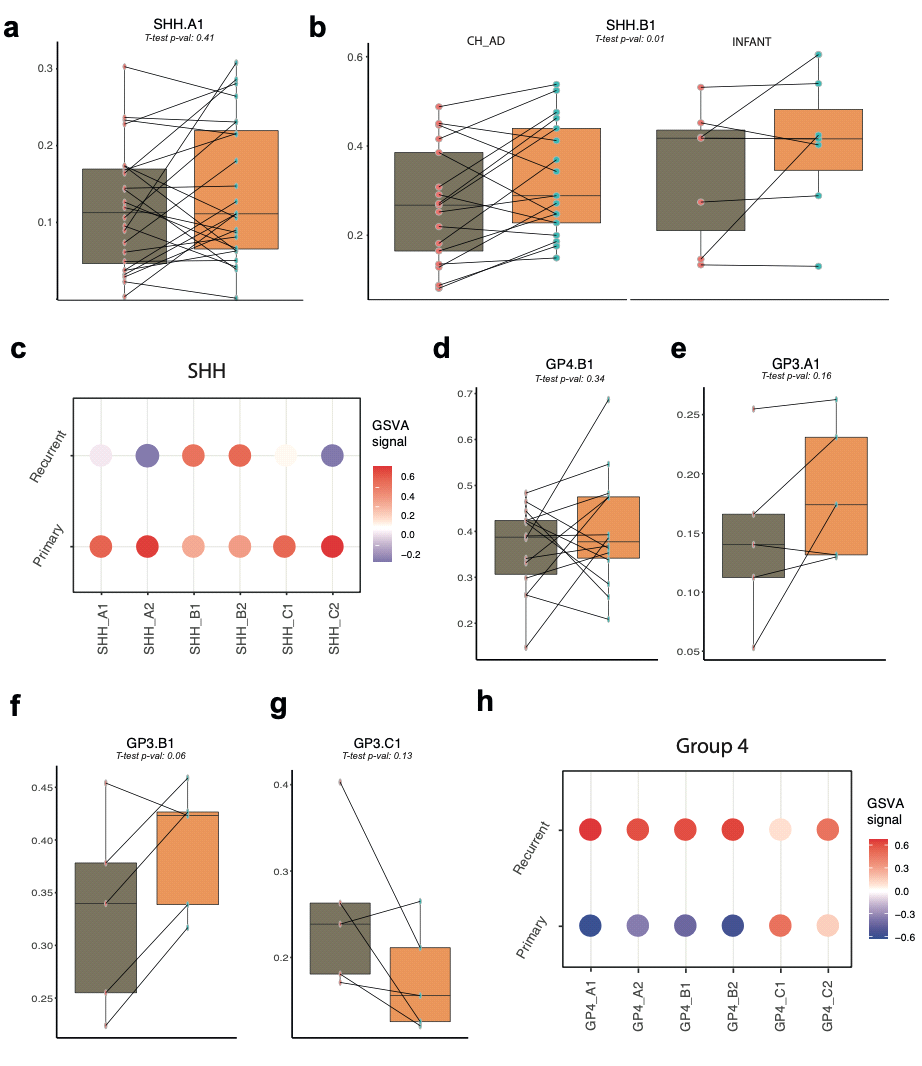 |
| --- |

***Suppl. Figure 6.*** *a) Boxplot of difference between MB SHH primary and relapse tumors in proportion of differentiated neuron-like cells C1. b) Boxplots of difference between primary and relapse tumors in proportions of undifferentiated progenitors B1 inspected for child-adult and infant SHH-MB subgroups separately. c) Gene set variance analysis (GSVA) applied on primary and relapse SHH_MB bulk transcriptome profiles focusing on corresponding cell type marker genes. d) Boxplot of difference between Group 4 MB primary and relapse tumors in proportion of undifferentiated progenitors B1 e,f,g) Boxplots of difference between Group 3 MB primary and relapse tumors in proportions of cell cycle enriched A1 (e), undifferentiated progenitors B1 (f) and differentiated neuron-like cells C1 (g). h) GSVA applied on primary and relapse Group 4 MB bulk transcriptome profiles focusing on corresponding cell type marker genes.*
